# Hexaaqua­manganese(II) tetra­aqua­bis(2-amino­pyrazine-κ*N* ^4^)manganese(II) disulfate dihydrate

**DOI:** 10.1107/S1600536809045322

**Published:** 2009-11-04

**Authors:** Li-Hua Huo, Shan Gao, Seik Weng Ng

**Affiliations:** a College of Chemistry and Materials Science, Heilongjiang University, Harbin 150080, People’s Republic of China; b Department of Chemistry, University of Malaya, 50603 Kuala Lumpur, Malaysia

## Abstract

The reaction of manganese(II) sulfate and 2-amino­pyrazine affords the title salt, [Mn(H_2_O)_6_][Mn(C_4_H_5_N_3_)_2_(H_2_O)_4_](SO_4_)_2_·2H_2_O. The metal atoms in the tetra­aqua-coordinated and hexa­aqua-coordinated cations lie on centers of inversion in octa­hedral geometries. The cations, anions and solvent water mol­ecules are linked by O—H⋯O, N—H⋯O and O—H⋯N hydrogen bonds into a three-dimensional network.

## Related literature

For the isostructural cobalt(II) analog, see: Kang *et al.* (2009 ▶).
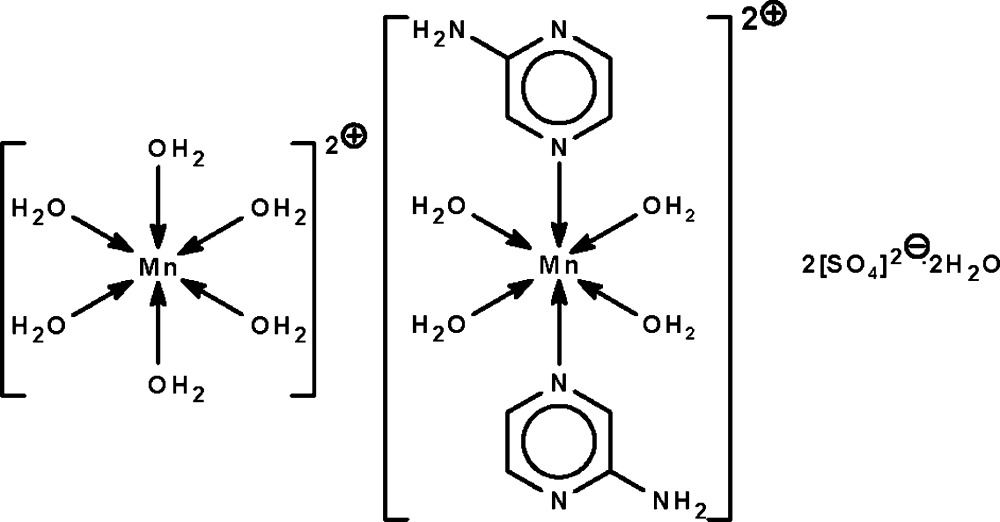


## Experimental

### 

#### Crystal data

[Mn(H_2_O)_6_][Mn(C_4_H_5_N_3_)_2_(H_2_O)_4_](SO_4_)_2_·2H_2_O
*M* *_r_* = 708.43
Triclinic, 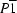

*a* = 6.6242 (3) Å
*b* = 8.4639 (4) Å
*c* = 13.2719 (8) Å
α = 75.654 (2)°
β = 78.364 (2)°
γ = 78.834 (2)°
*V* = 697.95 (6) Å^3^
*Z* = 1
Mo *K*α radiation
μ = 1.14 mm^−1^
*T* = 293 K
0.38 × 0.20 × 0.18 mm

#### Data collection

Rigaku R-AXIS RAPID IP diffractometer
Absorption correction: multi-scan (*ABSCOR*; Higashi, 1995 ▶) *T*_min_ = 0.670, *T*_max_ = 0.821
6866 measured reflections
3159 independent reflections
2874 reflections with *I* > 2σ(*I*)
*R*_int_ = 0.025

#### Refinement

*R*[*F* ^2^ > 2σ(*F* ^2^)] = 0.041
*wR*(*F* ^2^) = 0.129
*S* = 1.15
3159 reflections
231 parameters
14 restraints
H atoms treated by a mixture of independent and constrained refinement
Δρ_max_ = 0.74 e Å^−3^
Δρ_min_ = −0.40 e Å^−3^

### 

Data collection: *RAPID-AUTO* (Rigaku, 1998 ▶); cell refinement: *RAPID-AUTO*; data reduction: *CrystalClear* (Rigaku/MSC, 2002 ▶); program(s) used to solve structure: *SHELXS97* (Sheldrick, 2008 ▶); program(s) used to refine structure: *SHELXL97* (Sheldrick, 2008 ▶); molecular graphics: *X-SEED* (Barbour, 2001 ▶); software used to prepare material for publication: *publCIF* (Westrip, 2009 ▶).

## Supplementary Material

Crystal structure: contains datablocks global, I. DOI: 10.1107/S1600536809045322/xu2658sup1.cif

Structure factors: contains datablocks I. DOI: 10.1107/S1600536809045322/xu2658Isup2.hkl

Additional supplementary materials:  crystallographic information; 3D view; checkCIF report

## Figures and Tables

**Table 1:** Hydrogen-bond geometry (Å, °)

*D*—H⋯*A*	*D*—H	H⋯*A*	*D*⋯*A*	*D*—H⋯*A*
O1w—H1w1⋯O1	0.84 (1)	1.95 (2)	2.779 (3)	167 (4)
O1w—H1w2⋯N2^i^	0.85 (1)	1.94 (1)	2.792 (3)	176 (5)
O2w—H2w1⋯O3	0.85 (1)	1.95 (1)	2.775 (3)	166 (4)
O2w—H2w2⋯O1^ii^	0.85 (1)	1.92 (1)	2.770 (3)	172 (4)
O3w—H3w1⋯O2	0.85 (1)	1.90 (1)	2.744 (4)	170 (5)
O3w—H3w2⋯O6w	0.85 (1)	1.88 (1)	2.728 (4)	175 (4)
O4w—H4w1⋯O6w^iii^	0.85 (1)	1.96 (2)	2.780 (4)	162 (5)
O4w—H4w2⋯O2^iii^	0.85 (1)	1.92 (2)	2.744 (4)	164 (5)
O5w—H5w1⋯O3^iv^	0.84 (1)	2.00 (2)	2.813 (3)	159 (5)
O5w—H5w2⋯O4^v^	0.85 (1)	1.88 (1)	2.726 (4)	177 (6)
O6w—H6w1⋯O3^i^	0.85 (1)	1.95 (2)	2.783 (3)	167 (6)
O6w—H6w2⋯O4^iv^	0.85 (1)	1.87 (1)	2.709 (4)	172 (6)
N3—H3n2⋯O1^vi^	0.85 (1)	2.18 (1)	3.026 (4)	172 (5)

Symmetry codes: (i) 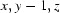; (ii) 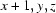; (iii) 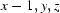; (iv) 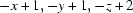; (v) 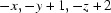; (vi) 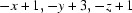.

**Fig. 1.**
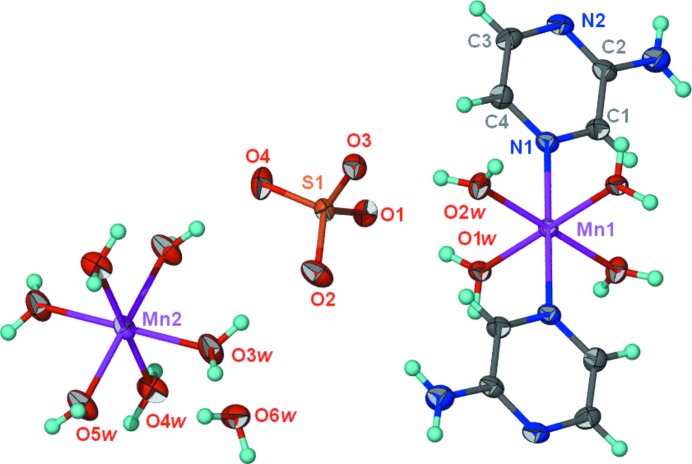
Thermal ellipsoid plot (Barbour, 2001) of [Mn(H2O)6] [Mn(H2O)4(C4H5N3)2] 2[SO4].2H2O at the 50% probability level; hydrogen atoms are drawn as spheres of arbitrary radius.

## supplementary crystallographic information

### Experimental

To an aqueous solution of 3-aminopyrazine (0.19 g, 2 mmol) was added manganese(II) sulfate tetrahydrate (0.45 g, 2 mmol). Colorless crystals of the salt separated from the solution after a few days. CH&N elemental analysis. Calc. for C_8_H_34_N_6_O_20_S_2_Mn_2_: C 13.56, H 4.84, N 11.86%; found: C 13.52, H 4.80, N 11.85%.

### Refinement

Carbon-bound H-atoms were placed in calculated positions (C—H 0.93 Å) and were included in the refinement in the riding model approximation, with *U*(H) set to 1.2*U*(C). The amino and water H-atoms were located in a difference Fourier map, and were refined with a distance restraint of N–H = O–H = 0.85±0.01 Å; their temperature factors were refined.

### Crystal data

**Table:**

[Mn(H_2_O)_6_][Mn(C_4_H_5_N_3_)_2_(H_2_O)_4_](SO_4_)_2_·2H_2_O	*Z* = 1
*M**_r_* = 708.43	*F*(000) = 366
Triclinic, *P*1	*D*_x_ = 1.685 Mg m^−^^3^
Hall symbol: -P 1	Mo *K*α radiation, λ = 0.71073 Å
*a* = 6.6242 (3) Å	Cell parameters from 6492 reflections
*b* = 8.4639 (4) Å	θ = 3.2–27.5°
*c* = 13.2719 (8) Å	µ = 1.14 mm^−^^1^
α = 75.654 (2)°	*T* = 293 K
β = 78.364 (2)°	Prism, colorless
γ = 78.834 (2)°	0.38 × 0.20 × 0.18 mm
*V* = 697.95 (6) Å^3^	

### Data collection

**Table:**

Rigaku R-AXIS RAPID IP diffractometer	3159 independent reflections
Radiation source: fine-focus sealed tube	2874 reflections with *I* > 2σ(*I*)
graphite	*R*_int_ = 0.025
ω scans	θ_max_ = 27.5°, θ_min_ = 3.2°
Absorption correction: multi-scan (*ABSCOR*; Higashi, 1995)	*h* = −8→8
*T*_min_ = 0.670, *T*_max_ = 0.821	*k* = −10→10
6866 measured reflections	*l* = −17→17

### Refinement

**Table:**

Refinement on *F*^2^	Primary atom site location: structure-invariant direct methods
Least-squares matrix: full	Secondary atom site location: difference Fourier map
*R*[*F*^2^ > 2σ(*F*^2^)] = 0.041	Hydrogen site location: inferred from neighbouring sites
*wR*(*F*^2^) = 0.129	H atoms treated by a mixture of independent and constrained refinement
*S* = 1.15	*w* = 1/[σ^2^(*F*_o_^2^) + (0.0556*P*)^2^ + 1.1503*P*] where *P* = (*F*_o_^2^ + 2*F*_c_^2^)/3
3159 reflections	(Δ/σ)_max_ = 0.001
231 parameters	Δρ_max_ = 0.74 e Å^−^^3^
14 restraints	Δρ_min_ = −0.40 e Å^−^^3^

### Fractional atomic coordinates and isotropic or equivalent isotropic displacement parameters (Å^2^)

**Table:**

	*x*	*y*	*z*	*U*_iso_*/*U*_eq_	
Mn1	1.0000	1.0000	0.5000	0.02349 (17)	
Mn2	0.0000	0.5000	1.0000	0.02867 (18)	
S1	0.47994 (10)	0.88750 (9)	0.80279 (5)	0.02457 (18)	
O1	0.4091 (3)	0.9828 (3)	0.70435 (17)	0.0322 (5)	
O2	0.5386 (4)	0.7129 (3)	0.7978 (2)	0.0444 (6)	
O3	0.6644 (3)	0.9501 (3)	0.81657 (17)	0.0318 (5)	
O4	0.3121 (4)	0.9084 (4)	0.89137 (19)	0.0441 (6)	
O1W	0.7084 (4)	0.9110 (3)	0.53580 (19)	0.0335 (5)	
O2W	1.0209 (4)	0.9617 (3)	0.66525 (17)	0.0345 (5)	
O3W	0.2937 (4)	0.4732 (3)	0.8939 (2)	0.0471 (7)	
O4W	−0.1480 (4)	0.4748 (3)	0.8707 (2)	0.0429 (6)	
O5W	0.0400 (4)	0.2353 (3)	1.0577 (2)	0.0456 (6)	
O6W	0.6189 (4)	0.2200 (3)	0.9081 (2)	0.0399 (6)	
N1	0.8363 (4)	1.2689 (3)	0.4980 (2)	0.0301 (5)	
N2	0.7098 (4)	1.6040 (3)	0.4930 (2)	0.0344 (6)	
N3	0.7810 (6)	1.6587 (4)	0.3104 (3)	0.0482 (8)	
C1	0.8414 (5)	1.3800 (4)	0.4078 (3)	0.0334 (7)	
H1	0.8899	1.3447	0.3450	0.040*	
C2	0.7760 (5)	1.5496 (4)	0.4039 (3)	0.0325 (6)	
C3	0.7019 (5)	1.4892 (4)	0.5836 (3)	0.0376 (7)	
H3	0.6532	1.5237	0.6466	0.045*	
C4	0.7623 (5)	1.3238 (4)	0.5874 (3)	0.0348 (7)	
H4	0.7522	1.2491	0.6521	0.042*	
H1W1	0.614 (5)	0.948 (5)	0.581 (3)	0.048 (12)*	
H1W2	0.715 (7)	0.817 (3)	0.521 (4)	0.053 (13)*	
H2W1	0.919 (4)	0.972 (5)	0.714 (2)	0.028 (9)*	
H2W2	1.135 (4)	0.967 (5)	0.683 (3)	0.044 (11)*	
H3W1	0.357 (7)	0.556 (4)	0.866 (4)	0.066 (15)*	
H3W2	0.391 (5)	0.392 (3)	0.901 (3)	0.039 (11)*	
H4W1	−0.195 (8)	0.386 (4)	0.876 (4)	0.073 (17)*	
H4W2	−0.247 (5)	0.554 (4)	0.859 (4)	0.063 (15)*	
H5W1	0.126 (6)	0.160 (4)	1.089 (3)	0.059 (14)*	
H5W2	−0.072 (5)	0.194 (7)	1.074 (5)	0.081 (18)*	
H6W1	0.624 (9)	0.148 (5)	0.873 (4)	0.079 (18)*	
H6W2	0.641 (9)	0.170 (6)	0.9698 (19)	0.072 (17)*	
H3N1	0.831 (7)	1.629 (6)	0.252 (2)	0.062 (15)*	
H3N2	0.738 (8)	1.7613 (19)	0.308 (4)	0.060 (14)*	

### Atomic displacement parameters (Å^2^)

**Table:**

	*U*^11^	*U*^22^	*U*^33^	*U*^12^	*U*^13^	*U*^23^
Mn1	0.0217 (3)	0.0225 (3)	0.0255 (3)	−0.0022 (2)	−0.0054 (2)	−0.0034 (2)
Mn2	0.0218 (3)	0.0271 (3)	0.0341 (3)	−0.0032 (2)	−0.0067 (2)	0.0002 (2)
S1	0.0209 (3)	0.0257 (3)	0.0260 (3)	−0.0046 (3)	−0.0066 (2)	−0.0004 (3)
O1	0.0289 (11)	0.0353 (12)	0.0302 (11)	−0.0007 (9)	−0.0115 (9)	−0.0009 (9)
O2	0.0423 (14)	0.0252 (11)	0.0645 (17)	−0.0035 (10)	−0.0175 (12)	−0.0014 (11)
O3	0.0224 (10)	0.0385 (12)	0.0356 (11)	−0.0062 (9)	−0.0079 (8)	−0.0062 (9)
O4	0.0313 (12)	0.0672 (17)	0.0317 (12)	−0.0132 (12)	0.0012 (10)	−0.0078 (11)
O1W	0.0277 (11)	0.0325 (12)	0.0423 (13)	−0.0092 (9)	0.0038 (9)	−0.0157 (10)
O2W	0.0258 (11)	0.0521 (14)	0.0264 (10)	−0.0058 (10)	−0.0080 (9)	−0.0068 (10)
O3W	0.0281 (12)	0.0356 (14)	0.0671 (18)	−0.0064 (11)	0.0055 (12)	−0.0012 (12)
O4W	0.0395 (14)	0.0380 (14)	0.0542 (15)	−0.0039 (11)	−0.0212 (12)	−0.0059 (12)
O5W	0.0318 (13)	0.0320 (12)	0.0679 (17)	−0.0074 (10)	−0.0194 (12)	0.0096 (12)
O6W	0.0477 (14)	0.0317 (12)	0.0413 (14)	−0.0016 (11)	−0.0131 (11)	−0.0086 (10)
N1	0.0269 (12)	0.0226 (12)	0.0390 (14)	−0.0011 (10)	−0.0063 (10)	−0.0044 (10)
N2	0.0293 (13)	0.0255 (12)	0.0498 (16)	−0.0013 (10)	−0.0095 (12)	−0.0109 (11)
N3	0.063 (2)	0.0285 (15)	0.0451 (18)	0.0031 (15)	−0.0061 (16)	−0.0039 (13)
C1	0.0347 (16)	0.0256 (15)	0.0380 (16)	0.0001 (12)	−0.0061 (13)	−0.0069 (12)
C2	0.0289 (15)	0.0238 (14)	0.0435 (17)	−0.0014 (12)	−0.0071 (13)	−0.0060 (12)
C3	0.0337 (16)	0.0391 (18)	0.0425 (18)	0.0019 (14)	−0.0073 (14)	−0.0182 (14)
C4	0.0318 (16)	0.0335 (16)	0.0352 (16)	0.0037 (13)	−0.0075 (13)	−0.0051 (13)

### Geometric parameters (Å, °)

**Table:**

Mn1—O1W	2.131 (2)	O3W—H3W1	0.849 (10)
Mn1—O1W^i^	2.131 (2)	O3W—H3W2	0.849 (10)
Mn1—O2W^i^	2.165 (2)	O4W—H4W1	0.848 (10)
Mn1—O2W	2.165 (2)	O4W—H4W2	0.850 (10)
Mn1—N1	2.320 (2)	O5W—H5W1	0.849 (10)
Mn1—N1^i^	2.320 (2)	O5W—H5W2	0.850 (10)
Mn2—O3W	2.168 (3)	O6W—H6W1	0.850 (10)
Mn2—O3W^ii^	2.168 (3)	O6W—H6W2	0.850 (10)
Mn2—O4W	2.212 (3)	N1—C1	1.325 (4)
Mn2—O4W^ii^	2.212 (3)	N1—C4	1.347 (4)
Mn2—O5W^ii^	2.163 (2)	N2—C2	1.337 (4)
Mn2—O5W	2.163 (2)	N2—C3	1.344 (5)
S1—O4	1.466 (2)	N3—C2	1.350 (5)
S1—O2	1.468 (3)	N3—H3N1	0.855 (10)
S1—O1	1.468 (2)	N3—H3N2	0.854 (10)
S1—O3	1.482 (2)	C1—C2	1.408 (4)
O1W—H1W1	0.844 (10)	C1—H1	0.9300
O1W—H1W2	0.851 (10)	C3—C4	1.371 (5)
O2W—H2W1	0.846 (10)	C3—H3	0.9300
O2W—H2W2	0.852 (10)	C4—H4	0.9300
			
O1W—Mn1—O1W^i^	180.000 (1)	Mn1—O1W—H1W1	118 (3)
O1W—Mn1—O2W^i^	87.90 (9)	Mn1—O1W—H1W2	114 (3)
O1W^i^—Mn1—O2W^i^	92.10 (9)	H1W1—O1W—H1W2	123 (4)
O1W—Mn1—O2W	92.10 (9)	Mn1—O2W—H2W1	125 (3)
O1W^i^—Mn1—O2W	87.90 (9)	Mn1—O2W—H2W2	120 (3)
O2W^i^—Mn1—O2W	180.000 (1)	H2W1—O2W—H2W2	112 (4)
O1W—Mn1—N1	91.71 (9)	Mn2—O3W—H3W1	120 (4)
O1W^i^—Mn1—N1	88.29 (9)	Mn2—O3W—H3W2	126 (3)
O2W^i^—Mn1—N1	89.32 (10)	H3W1—O3W—H3W2	104 (4)
O2W—Mn1—N1	90.68 (10)	Mn2—O4W—H4W1	119 (4)
O1W—Mn1—N1^i^	88.29 (9)	Mn2—O4W—H4W2	110 (3)
O1W^i^—Mn1—N1^i^	91.71 (9)	H4W1—O4W—H4W2	107 (5)
O2W^i^—Mn1—N1^i^	90.68 (10)	Mn2—O5W—H5W1	139 (3)
O2W—Mn1—N1^i^	89.32 (10)	Mn2—O5W—H5W2	115 (4)
N1—Mn1—N1^i^	180.000 (1)	H5W1—O5W—H5W2	103 (5)
O5W^ii^—Mn2—O5W	180.000 (1)	H6W1—O6W—H6W2	108 (5)
O5W^ii^—Mn2—O3W	90.64 (10)	C1—N1—C4	117.4 (3)
O5W—Mn2—O3W	89.36 (10)	C1—N1—Mn1	119.9 (2)
O5W^ii^—Mn2—O3W^ii^	89.36 (10)	C4—N1—Mn1	121.9 (2)
O5W—Mn2—O3W^ii^	90.64 (10)	C2—N2—C3	116.8 (3)
O3W—Mn2—O3W^ii^	180.000 (1)	C2—N3—H3N1	122 (3)
O5W^ii^—Mn2—O4W	89.84 (10)	C2—N3—H3N2	121 (3)
O5W—Mn2—O4W	90.16 (10)	H3N1—N3—H3N2	117 (5)
O3W—Mn2—O4W	86.45 (11)	N1—C1—C2	122.2 (3)
O3W^ii^—Mn2—O4W	93.55 (11)	N1—C1—H1	118.9
O5W^ii^—Mn2—O4W^ii^	90.16 (10)	C2—C1—H1	118.9
O5W—Mn2—O4W^ii^	89.84 (10)	N2—C2—N3	119.4 (3)
O3W—Mn2—O4W^ii^	93.55 (11)	N2—C2—C1	120.2 (3)
O3W^ii^—Mn2—O4W^ii^	86.45 (11)	N3—C2—C1	120.4 (3)
O4W—Mn2—O4W^ii^	180.000 (1)	N2—C3—C4	123.1 (3)
O4—S1—O2	110.85 (17)	N2—C3—H3	118.4
O4—S1—O1	109.03 (14)	C4—C3—H3	118.4
O2—S1—O1	109.60 (15)	N1—C4—C3	120.3 (3)
O4—S1—O3	109.10 (14)	N1—C4—H4	119.9
O2—S1—O3	109.02 (14)	C3—C4—H4	119.9
O1—S1—O3	109.22 (13)		

Symmetry codes: (i) −*x*+2, −*y*+2, −*z*+1; (ii) −*x*, −*y*+1, −*z*+2.

### Hydrogen-bond geometry (Å, °)

**Table:**

*D*—H···*A*	*D*—H	H···*A*	*D*···*A*	*D*—H···*A*
O1w—H1w1···O1	0.84 (1)	1.95 (2)	2.779 (3)	167 (4)
O1w—H1w2···N2^iii^	0.85 (1)	1.94 (1)	2.792 (3)	176 (5)
O2w—H2w1···O3	0.85 (1)	1.95 (1)	2.775 (3)	166 (4)
O2w—H2w2···O1^iv^	0.85 (1)	1.92 (1)	2.770 (3)	172 (4)
O3w—H3w1···O2	0.85 (1)	1.90 (1)	2.744 (4)	170 (5)
O3w—H3w2···O6w	0.85 (1)	1.88 (1)	2.728 (4)	175 (4)
O4w—H4w1···O6w^v^	0.85 (1)	1.96 (2)	2.780 (4)	162 (5)
O4w—H4w2···O2^v^	0.85 (1)	1.92 (2)	2.744 (4)	164 (5)
O5w—H5w1···O3^vi^	0.84 (1)	2.00 (2)	2.813 (3)	159 (5)
O5w—H5w2···O4^ii^	0.85 (1)	1.88 (1)	2.726 (4)	177 (6)
O6w—H6w1···O3^iii^	0.85 (1)	1.95 (2)	2.783 (3)	167 (6)
O6w—H6w2···O4^vi^	0.85 (1)	1.87 (1)	2.709 (4)	172 (6)
N3—H3n2···O1^vii^	0.85 (1)	2.18 (1)	3.026 (4)	172 (5)

Symmetry codes: (iii) *x*, *y*−1, *z*; (iv) *x*+1, *y*, *z*; (v) *x*−1, *y*, *z*; (vi) −*x*+1, −*y*+1, −*z*+2; (ii) −*x*, −*y*+1, −*z*+2; (vii) −*x*+1, −*y*+3, −*z*+1.
